# Osteopontin is a multi-faceted pro-tumorigenic driver for central nervous system lymphoma

**DOI:** 10.18632/oncotarget.8537

**Published:** 2016-04-01

**Authors:** Qiu Yushi, Zhimin Li, Christina A. Von Roemeling, Heike Doeppler, Laura A. Marlow, Betty Y.S. Kim, Derek C. Radisky, Peter Storz, John A. Copland, Han W. Tun

**Affiliations:** ^1^ Department of Cancer Biology, Jacksonville, Florida, USA; ^2^ Mayo Graduate School, Mayo Clinic, Rochester, Minnesota, USA; ^3^ Department of Neurosurgery, Mayo Clinic, Jacksonville, Florida, USA; ^4^ Department of Hematology/Oncology, Mayo Clinic, Jacksonville, Florida, USA

**Keywords:** osteopontin (OPN), CNS lymphoma, proliferation, invasion, NF-κB signaling

## Abstract

Osteopontin (OPN) is the most upregulated gene in primary central nervous system lymphoma (PCNSL) compared to non-CNS diffuse large B cell lymphoma (DLBCL). We show here that OPN is a key mediator of intracerebral tumor growth, invasion, and dissemination in CNS lymphoma, and that these effects depend upon activation of NF-κB. We further show that activation of NF-κB by OPN occurs through a unique mechanism in which intracellular OPN (iOPN) causes transcriptional downregulation of the NF-κB inhibitors, A20/TNFAIP3 and ABIN1/TNIP1, and secretory OPN (sOPN) promotes receptor-mediated activation of NF-κB. We also identify NF-κB-mediated induction of matrix metalloproteinase-8 (MMP-8) as a specific feature of OPN-mediated tissue invasion. These results implicate OPN as a candidate for development of targeted therapy for patients with PCNSL.

## INTRODUCTION

Primary CNS lymphoma (PCNSL) is a DLBCL confined to the CNS [1–3]. It accounts for 3–4% of brain tumors [4]. Biologically, PCNSL is enigmatic as there are very few resident B lymphocytes in the CNS under normal circumstances [5]. PCNSL has been called a “whole brain disease” as autopsy studies frequently reveal widespread intracerebral dissemination of lymphoma cells, even in areas of brain which appeared normal on imaging scans [6]. PCNSL lymphoma cells exhibit highly selective CNS tropism [7], an operative trait that is likely critical in the pathogenesis of PCNSL.

The survival of patients with PCNSL has improved with the development of combination induction therapy and the incorporation of intensive consolidation chemotherapy, whole brain radiation, or high-dose chemotherapy followed by autologous stem cell transplantation [8]. However, these treatments are toxic and are not well tolerated especially by elderly patients who make up the majority of immunocompetent patients with PCNSL [2, 3]. Moreover, survival is expected to plateau soon with currently available therapeutic agents [8]. As such, there is a dire need for novel therapeutic agents.

In an effort to identify tumor-specific drivers of disease pathogenesis, our group previously performed a genome-wide gene expression analysis comparing PCNSL to non-CNS DLBCL. The results revealed a “CNS signature” of PCNSL, characterized by unique differential expression of genes and pathways [9]. We found that osteopontin (OPN, *SPP1*) is the most upregulated gene (~10 fold) in PCNSL compared to non-CNS DLBCL. This finding has since been corroborated by others [10], and OPN over-expression has also been reported in an aggressive subset of systemic DLBCL with poor prognosis [11]. OPN has been implicated in a variety of biological processes such as extracellular matrix (ECM) adhesion and remodeling, cytokine activity, immune regulation, stress response, cell migration, angiogenesis, and proliferation [12–14]. Dysregulation of OPN is linked to a number of diseases including autoimmune disorders, inflammatory diseases, fibrosis, and malignancy [14, 15]. In the immune system, OPN behaves as a multifunctional cytokine, and is produced by B lymphocytes and also promotes B cell proliferation and immunoglobulin production [14, 16]. While OPN is expressed at low levels by normal brain cells [17], increased expression has been observed in CNS diseases such as multiple sclerosis [18] and primary brain tumors such as glioblastoma multiforme and astrocytoma [19, 20]. Currently, a specific role for OPN in PCNSL has not been defined.

In this study, we perform functional genomic analysis to dissect the role of OPN in CNSL using *in vitro, ex vivo*, and *in vivo* experimental models. Our results demonstrate that OPN stimulates CNSL tumor cell proliferation, tumor growth, brain tissue invasiveness and intracerebral dissemination through a novel mechanism whereby OPN activates NF-κB activity via a novel dual mechanism in which intracellular OPN (iOPN) transcriptionally downregulates endogenous inhibitors of NF-ĸB, and secretory OPN (sOPN) directly activates NF-κB via an autocrine loop.

## RESULTS

### Osteopontin significantly increases proliferation and brain tissue invasiveness of B lymphoma cells

PCNSL is defined as a DLBCL confined to the CNS [1]. There has been no cell line derived from human PCNSL tissue. In order to investigate the functional role of OPN in PCNSL we employed two established DLBCL cell lines, OciLy3 and Rck8, as well as Raji cells, a Burkitt lymphoma cell line. Based on our screening of B lymphoma cells for OPN expression, OciLy3 and Rck8 cells demonstrate high levels of expression, while Raji cells demonstrate low level of endogenous OPN expression. To define the role of OPN in lymphoma cell phenotype, we used a targeted shRNA lentiviral construct against OPN (shRNA-OPN) to evaluate the effects of reduced OPN expression in OciLy3 and Rck8 cells, and an OPN expression construct (OPN) was transfected into Raji cells to evaluate the effects of OPN overexpression. Of note, OPN expression in OciLy3 cells could be only transiently knocked down.

**Figure 1:**
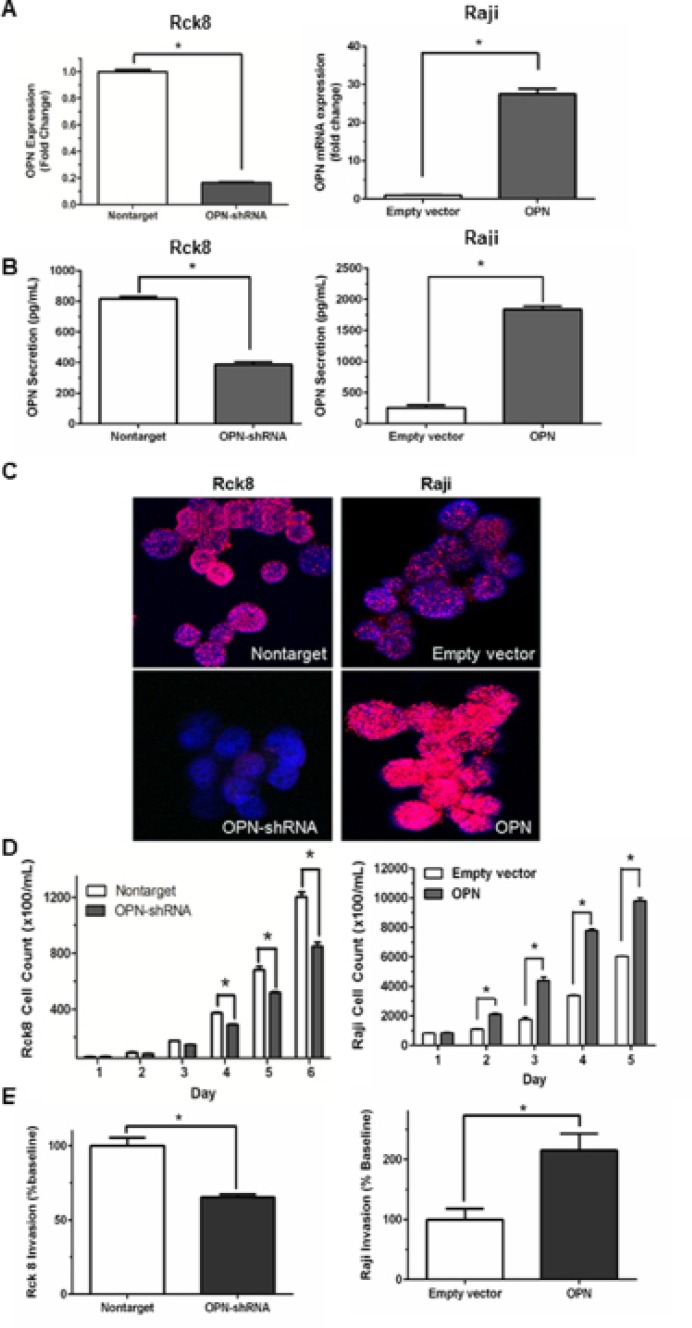
Osteopontin significantly increases B lymphoma cell proliferation and invasion Transfection experiments are performed to knock in and known down OPN in B lymphoma cells. OPN is knocked down in Rck8 cells (Rck8-shRNA) using lentiviral plasmid OPN shRNA and overexpressed in Raji cells (Raji-OPN) using lentiviral plasmid with OPN. The impact of OPN on the B lymphoma cells is analyzed by cell proliferation and Transwell invasion assays. (**A**) Q-PCR quantification for OPN mRNA level in the Rck8 OPN knock-down (OPN-shRNA) and Raji OPN overexpressing (OPN) cells compared to their controls. (**B**) ELISA assay for measurement of secretory OPN (sOPN) secreted into the culture media by the transfected B lymphoma cells compared to their controls. (**C**) Immunofluorescence (IF) for OPN protein expression in the transfected B lymphoma cells compared to their controls. Final original magnification, X 600 oil. (**D**) Cell proliferation assay of the transfected B lymphoma cells over a 5–6 day time course compared to respective controls. (**E**) Transwell invasion assay of the transfected B lymphoma cells compared to respective controls. (Error bars in the figures show SEM of triplicates. *P < 0.001).

qRT-PCR demonstrated an approximately 80% OPN knockdown in Rck8 OPN-shRNA cells and a nearly 28-fold OPN overexpression in Raji OPN transfected cells (Figure 1A). OPN can be localized to extracellular (secreted OPN, sOPN) and intracellular compartments (intracellular OPN, iOPN) [21]. ELISA assay for secreted OPN (sOPN) in the culture media showed decreased levels with Rck8 OPN-shRNA cells and increased levels with Raji OPN cells, as compared to respective controls (Figure 1B). In addition, immunofluorescence (IF) for OPN protein demonstrated a decrease in intracellular OPN (iOPN) in Rck8 OPN-shRNA cells, and an increase in iOPN in Raji OPN cells (Figure 1C). Thus, the OPN knockdown and knockin had an impact on both sOPN and iOPN in the lymphoma cells. Analysis of tumor cell proliferation showed that loss of OPN significantly attenuated proliferation in Rck8 OPN-shRNA, but increased proliferation in Raji cells overexpressing OPN (Figure 1D). Invasive capabilities in Rck8 and Raji clones were assessed by transwell invasion assay, and Rck8 OPN-shRNA cells exhibited decreased invasion while Raji-OPN cells demonstrated increased invasion as compared to respective controls (Figure 1E).

**Figure 2:**
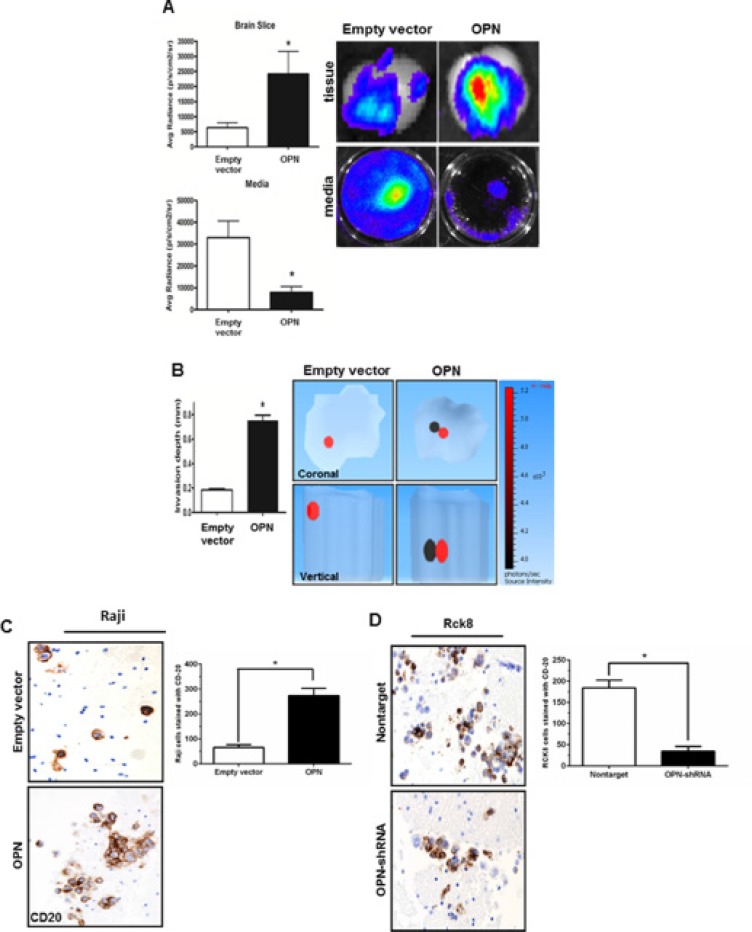
Osteopontin significantly increases the brain tissue invasiveness of B lymphoma cells The impact of OPN on invasiveness of B lymphoma cells in the brain tissue is studied by *ex vivo* murine brain slice invasion assay. Rck8-OPN shRNA and Raji-OPN cells were compared to their respective controls. (**A**) Bioluminescence imaging is used to analyze the invasion of luciferase-transfected Raji-OPN cells into murine brain slices compared to their respective control cells. OPN is shown to significantly promote the invasion of lymphoma cells into the brain slices. (**B**) Tomographic analysis is performed to analyze the invasion depth of the luciferase-transfected Raji-OPN B lymphoma cells into the murine brain slices compared to their controls. OPN is shown to significantly increase the depth of invasion. The dark circle indicates the most concentrated area of tumor cells and the red circle indicates the least concentrated area of tumor cells. The scale bar indicates the source intensity. (**C** and **D**) CD20 immunohistochemistry (IHC) is performed to identify the number of Raji-OPN and Rck8-OPN shRNA invading the brain slices compared to controls. OPN is shown to increase the number of B lymphoma cells invading the brain slices. (Error bars in the figures show SEM of triplicates. *P < 0.001).

Next, the invasive potential of Raji cells was further assessed *ex vivo* using a modified mice brain slice invasion assay [22]. Raji OPN overexpressing and control cells were infected with a lentiviral luciferase construct prior to assay. After 5 days of incubation, the extent of lymphoma cell invasion into the brain slice was quantitated by bioluminescence (average radiance) in both the brain tissue slice and media. Raji OPN cells demonstrated amplified brain tissue invasion as evidenced by considerably increased luminescent activity in the brain slice and decreased luminescent activity in the culture media (Figure 2A). 3D luminescent topography reconstruction showed deeper invasion of Raji-OPN cells into the brain slice compared to control cells, indicative of increased invasive capabilities in these cells (Figure 2B). Brain slices from the *ex vivo* invasion assay were then formalin-fixed, sectioned, and immunohistochemistry staining for CD20 was performed. A significant increase in the number of CD20+ B-cells was observed in brain tissue slices incubated with Raji-OPN cells (Figure 2C). The *ex vivo* brain slice assay was also performed using Rck8 non-target and OPN-shRNA cells. Unfortunately, luciferase could not be expressed in Rck8 cells, negating their use for experiments involving bioluminescent imaging. CD20 staining of fixed brain sections revealed that significantly fewer CD20+ Rck8 OPN-shRNA cells were able to penetrate into the brain tissue as compared to the nontarget control cells (Figure 2D).

### Osteopontin significantly increases intracerebral tumor growth, invasion, and dissemination of B lymphoma cells, shortening survival in athymic mice

**Figure 3:**
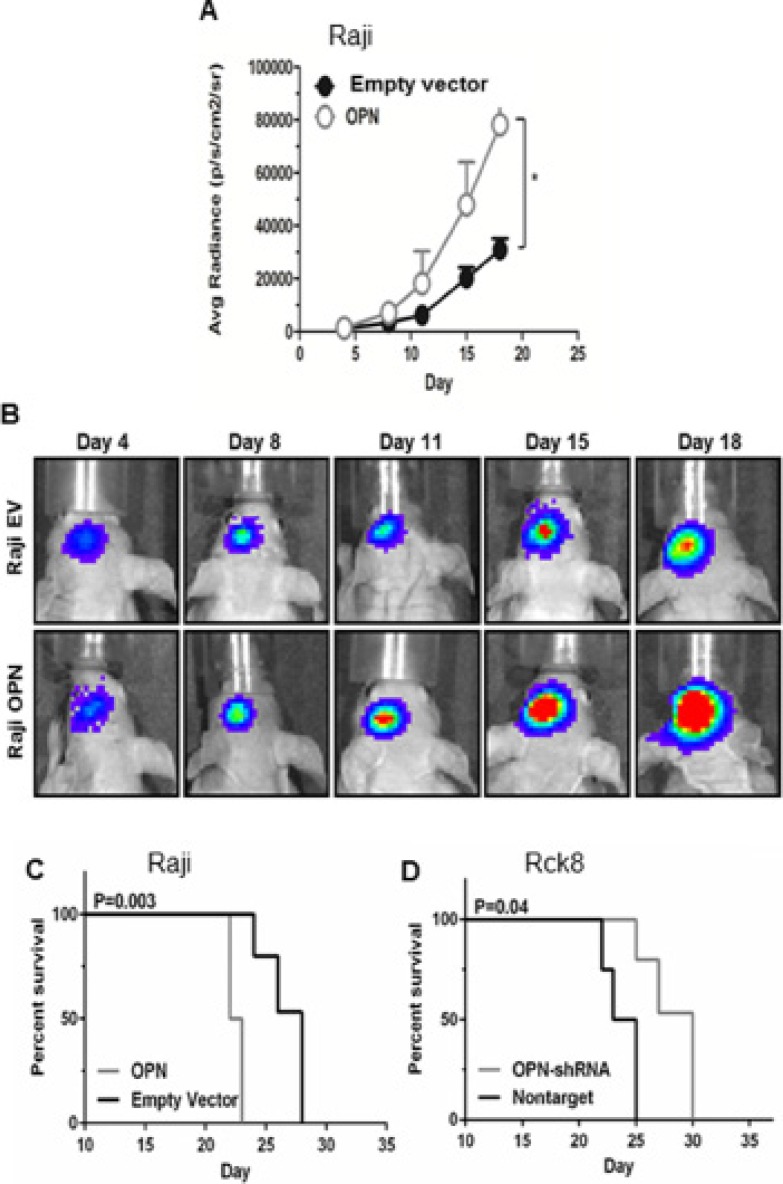
Osteopontin significantly increases the intracerebral lymphoma growth and shortens the survival in athymic mice Luciferase-transfected Raji-OPN lymphoma cells (2.5 × 10^4^) were intracerebrally implanted in athymic mice in the left periventricular area to analyse the impact of OPN on the intracerebral lymphoma growth as reflected by bioluminescence activity and survival, compared to their respective controls. Rck8 cells could not be stably transfected with luciferase. Rck8-OPN shRNA cells were intracerebrally implanted in athymic mice to study the impact of OPN on the survival, compared to their control. (**A** and **B**) Intracerebral implantation of Raji-OPN and Raji-EV control in athymic mice, showing a significant increase in intracerebral lymphoma growth with OPN overexpression. (In figure A, error bars show SEM of *N* = 9, *P < 0.05). (**C**, **D**) Kaplan Meier analysis shows a significant decrease in survival with OPN overexpression (Raji-OPN vs Raji-EV, C) and a significant increase in survival with OPN knock-down (Rck8-OPN shRNA vs Rck8-NT, D).

To evaluate the *in vivo* impact of OPN on intracerebral growth and dissemination of B lymphoma cells, we performed intracerebral implantation experiments using athymic mice. Raji-OPN, Rck8-shRNA, and their control cells were injected into the left periventricular area of the brain. Tumor growth and intracerebral dissemination of lymphoma cells to the contralateral (right) lobe of the brain were evaluated. As stated previously, Rck8 cells could not be stably transfected with luciferase and as a result tumor growth could not be monitored real-time *in vivo*; however survival analysis and intracerebral lymphoma dissemination were assessed. Raji-OPN cells revealed accelerated tumor growth as compared to empty vector control (Figure 3A–3B). Kaplan-Meier assessment, using limb paralysis as the end point, demonstrated reduced survival in Raji-OPN mice compared to empty vector controls (Figure 3C). Conversely, enhanced survival was observed in mice bearing tumors derived from Rck8 OPN-shRNA (Figure 3D) cells as compared to nontarget control.

**Figure 4:**
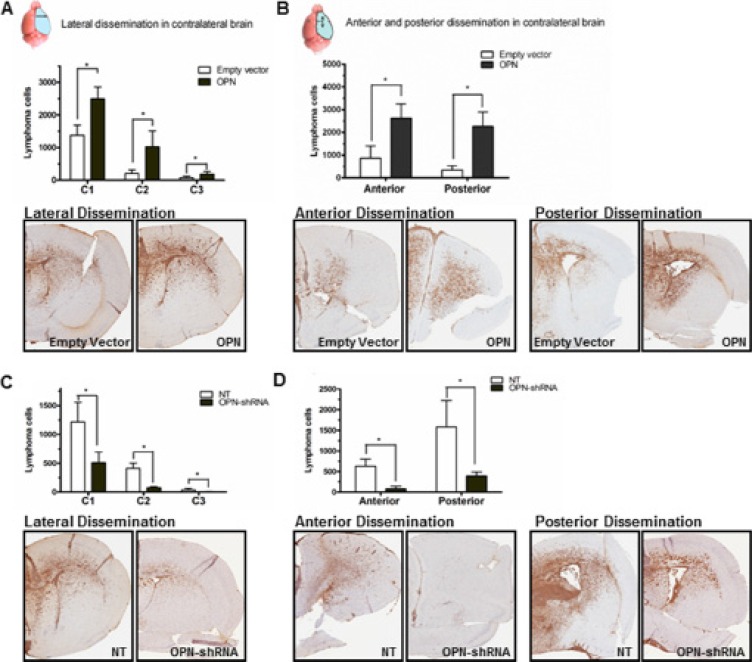
Osteopontin significantly promotes intracerebral inavasion and dissemination of lymphoma cells Murine brains were harvested after the animals expired in the intracerebral implantation experiment. Intracerebral dissemination of B lymphoma cells was determined by CD20 immunohistochemistry (IHC). Dissemination of lymphoma cells from the implanted site (left periventricular region of the brain) to contralateral (right) lobe of the brain in lateral, anterior, and posterior directions was determined by counting CD20+ lymphoma cells by Aperio CS2 image capture device. To determine the lateral spread, lymphoma cells were counted in three equal-sized compartments from the midline in the right lateral direction. For anterior and posterior dissemination, lymphoma cells were counted in the right anterior and posterior brain slices (Bregma 2.0 ± 0.3 mm; Bregma0.26 ± 0.3 mm). (**A** and **B**) Intracerebral dissemination of Raji-OPN cells is significantly increased in all directions compared to Raji-EV control cells. (**C** and **D**) Intracerebral dissemination of Rck8-OPN shRNA cells is significantly reduced in all directions compared to Rck8-NT control cells (*P* < 0.05).

PCNSL has been called a “whole brain disease” [6]. To ascertain the impact of OPN on the “whole brain nature” of CNS lymphoma, we performed CD20 IHC analysis in harvested murine brains. We quantitated dissemination of lymphoma cells in the contralateral (right) lobe in lateral, anterior, and posterior directions by counting the CD20+ lymphoma cells using Aperio CS2 image capture device. Quantitative analysis of directly lateral dissemination of lymphoma cells from the implantation site in left lobe of the brain into the right lobe was divided into 3 areas, where C1 represents lymphoma cell spreading into the right lobe directly lateral and adjacent to the injection site (medial third of R lobe), C2 represents cell distribution to the central third of the right lobe, and C3 represents spreading in the lateral exterior third of the right lobe. Raji-OPN cells demonstrated significantly increased invasion and dissemination into the contralateral lobe in all directions compared to empty vector control cells (Figure 4A and 4B). Rck8 OPN-shRNA cells demonstrated decreased invasion and dissemination in all directions (Figure 4C and 4D) compared to nontarget control cells.

### Osteopontin activates NF-κB signaling via a novel dual mechanism in which iOPN transcriptionally downregulates A20/TNFAIP3 and ABIN1/TNIP1 and sOPN acts on cell surface receptors via an autocrine loop

OPN has been linked to NF-κB mediated cell migration and invasion in a number of other cell types and malignancies [13, 23–25]. There are two main types of OPN based on the localization – secretory OPN (sOPN) and intracellular OPN (iOPN) [21]. Previous studies on OPN-mediated NF-κB activation have focused on sOPN acting as an intermediary for cell-ECM (extracellular matrix) interaction and binding to cell surface receptors such as integrins and CD44, resulting in IKK activation and NF-κB signaling [23–25].

**Figure 5:**
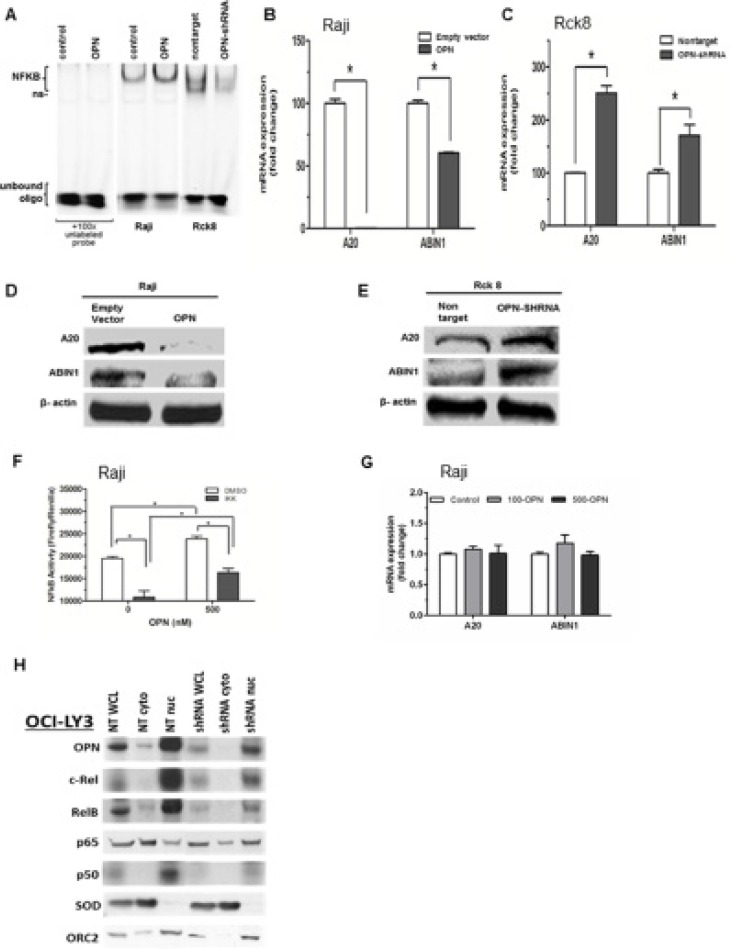
Osteopontin activates NF-κB signaling by a dual mechanism consisting of transcriptional downregulation of A20/TNFAIP3 and ABIN1/TNIP1 by intracellular OPN (iOPN) and an autocrine stimulation of cell membrane receptors by secretory OPN (sOPN) (**A**) Nuclear extracts of Raji-OPN (labeled OPN) and Rck8-OPN shRNA cells were analyzed with Electrophoretic Mobility Shift Assay (EMSA) compared to their respective controls, showing that OPN increased NF-κB DNA binding activity (ns = non-specific). (**B** and **C**) Q-PCR was performed on Raji-OPN and Rck8-OPN shRNA cells compared to their respective controls to analyze the expression of A20 and ABIN1 mRNA. OPN was shown to downregulate A20 and ABIN1. (**D** and **E**) Western blot analysis validated Q-PCR findings showing that OPN decreased A20 and ABIN1 expression. (**F** and **G**) Treatment of Raji cells with recombinant OPN increased NF-κB activity as shown by the reporter assay. NF-κB activity in OPN treated and untreated Raji cells were reduced by treatment with IKK2 inhibitor. Treatment of Raji cells with recombinant OPN at 100 (100-OPN) and 500 (500-OPN) nM did not result in any significant change in expression of A20 and ABIN1 mRNA as shown by Q-PCR. (**H**) Western analysis of OPN and NF-κB signaling proteins in cellular fractions (WCL- whole cell lysate; cyto – cytoplasmic fraction; nuc – nuclear fraction) of OCILY3 cells with OPN knockdown compared to nontarget (NT) controls. OPN knockdown is shown to decrease NF-κB activity as shown by changes in NF-κB signaling proteins. Cu/Zn Superoxide Dismutase (SOD), a cytoplasmic protein, and Origin Recognition Complex Subunit 2 (ORC2), a resident nuclear protein, were included to demonstrate successful cellular fractionation.

Constitutive activation of NF-κB is a key feature of PCNSL pathogenesis, driving tumor cell survival, proliferation, invasion, and protumorigenic inflammatory responses [26, 27]. However, a potential role for OPN in NF-κB activation in lymphoma cells has not been investigated We assessed the activity of nuclear NF-κB activity in response to OPN overexpression or knockdown by EMSA assay. Raji OPN cells yielded increased nuclear NF-κB activity, where OPN knockdown in Rck8 OPN-shRNA cells showed decreased nuclear NF-κB activity (Figure 5A). To examine the impact of OPN on regulatory mechanism of NF-κB signaling, we looked at A20/TNFAIP3 and ABIN1/TNIP1. Raji OPN cells showed significantly decreased expression of A20 and ABIN1 by QPCR and western compared to empty vector controls (Figure 5B and 5D). On the other hand, OPN knockdown in Rck8 cells led to a robust increase in expression of A20 and ABIN1 compared to nontarget controls (Figure 5C and 5E).

As our transfected Raji and Rck8 cells express iOPN as well as sOPN, we sought to examine their impact on NF-κB signaling separately. To define the impact of sOPN specifically on NF-κB signaling, we treated Raji cells with recombinant OPN (Figure 5F and 5G). It resulted in activation of NF-κB signaling as shown by the reporter assay without any significant change in A20 and ABIN1. These findings suggest that iOPN, not sOPN, mediates transcriptional downregulation of A20 and ABIN1. As such, OPN likely activates NF-κB signaling via two pathways: sOPN activates NF-κB signaling by acting on cell surface receptors via an autocrine loop and iOPN activates NF-κB signaling by transcriptional downregulation of A20 and ABIN1.

OCI-LY3 cells are human DLBCL cells with constitutive activation of NF-κB. To further validate our findings in Raji and Rck8 cells, we performed knockdown of OPN in OciLy3 cells by shRNA. To study the cellular localization of NF-κB transcription factors, cellular fractionation was performed, and distribution of OPN and NF-κB subunits was evaluated by western (Figure 5H). It is noteworthy that OPN is predominantly localized to the nuclear fraction just like in PCNSL as previously indicated by IHC [9]. Our results showed that knockdown of OPN led to suppressed NF-κB activity as revealed by decreased nuclear accumulation of canonical NF-κB factors including c-Rel, Rel-B, and p50 as compared to NT control cells.

### Osteopontin promotes B lymphoma cell proliferation and invasion via NF-κB activation

**Figure 6:**
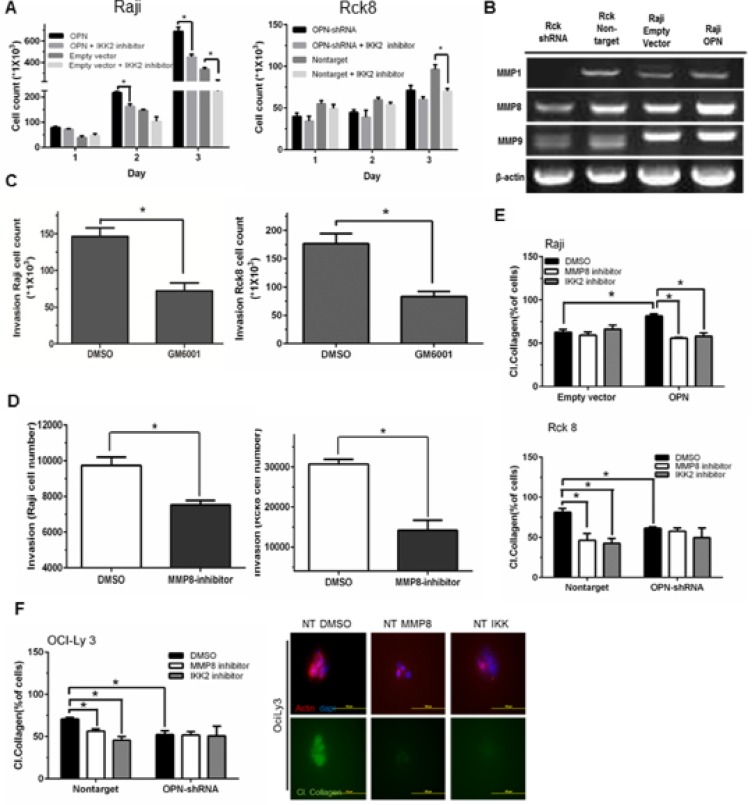
Osteopontin promotes B lymphoma cell proliferation and MMP8-mediated invasion of B lymphoma cells via activation of NF-κB signaling (**A**) Raji-OPN cells, Rck8-OPN shRNA, and their control cells were treated with IKK2 inhibitor. OPN-mediated increase in cell proliferation is shown to be negated by IKK2 inhibitor. (**B**) RT-PCR analysis of MMPs in Raji-OPN, Rck8-OPN shRNA, and their control cells showing that OPN increased MMP8 expression concordantly in both cell models. (**C** and **D**) Transwell invasion assay showing decreased invasion by Raji and Rck8 cells with Pan-MMP or MMP8 inhibition. (**E** and **F**) Collagenase assay on Raji-OPN, Rck8-OPN shRNA cells, and OCILY3-OPN shRNA cells, and their respective control cells treated with DMSO, MMP8 inhibitor, or IKK2 inhibitor, showing that OPN-mediated MMP8 activity (collagenase activity) is negated by MMP8 or NF-κB inhibition. The confocal microscopy on OCILY3 nontarget cells shows that collagenase activity (green) is eliminated by inhibition of MMP8 or NF-κB inhibition. Phalloidin (red) and Dapi (blue) stains were used to locate lymphoma cells.). Representative images depicting collagen cleavage in response to DMSO control, MMP8 inhibitor and IKK 2 inhibitor are shown.

As OPN activates NF-κB signaling, we next examined whether the phenotypic observations resulting from OPN expression were mediated through NF-κB signaling. Tumor cell proliferation in response to OPN overexpression in Raji cells was significantly attenuated when these cells were treated with the NF-κB inhibitor (IKK2 inhibitor) (Figure 6A), while there was a lesser decrease in proliferation of empty vector control cells treated with IKK2 inhibitor (Figure 6A). Rck8 cells were also treated with the NF-κB inhibitor (IKK2 inhibitor) (Figure 6A). A significant attenuation of proliferation of Rck8 NT control cells was seen with IKK2 inhibitor whereas a similar effect was not seen with Rck8 shRNA cells (Figure 6A). All these results suggest that OPN-driven cell proliferation is mediated via NF-κB activation. NF-κB has been correlated with extracellular matrix degradation and cellular invasion via regulation of various matrix metalloproteinases (MMPs) [28, 29]. Because OPN expression increases lymphoma cell invasiveness both *in vitro, ex vivo*, and *in vivo*, we examined the impact of OPN expression on the differential expression of MMPs in Raji and Rck8 cells. Decreased levels of MMP1, MMP8, and MMP9 mRNA were observed by RT-PCR in Rck8 OPN-shRNA cells; however only MMP8 levels appeared to be concordantly induced by OPN overexpression in Raji- OPN cells (Figure 6B). Invasiveness of Raji and Rck8 cells in response to a pan-MMP inhibitor (GM6001) was examined by transwell invasion assay. GM6001 (10 uM) significantly decreased the number of invading cells (Figure 6C). As OPN expression correlated most with MMP8 in both lymphoma cell models, we further examined the role of MMP8 in lymphoma cell invasion using a specific inhibitor of MMP8 in transwell invasion assays. Invasiveness of both cell lines was significantly attenuated with inhibition of MMP8 (Figure 6D). Since MMP8 functions primarily as a collagenase, we examined the effects of OPN expression on the ability of lymphoma cells to degrade collagen. Cells were suspended in 3D in a mixture of matrigel and fluorescein-conjugated collagen type I, and cultured in growth medium containing DMSO (control), MMP8 inhibitor (10 uM), or IKK-2 inhibitor (10 uM). Cleavage of collagen resulted in increased fluorescein fluorescent signal, indicative of collagenase activity. Raji OPN cells exhibited increased collagenase activity as compared to empty vector control cells, and this was blocked with the addition of either the MMP8 or IKK-2 inhibitor (Figure 6E). Rck8 nontarget cells demonstrated significantly more collagenase activity as compared to OPN-shRNA cells (Figure 6E). This was significantly decreased with either MMP8 or IKK-2 inhibitor treatment (Figure 6E). MMP8 and IKK-2 inhibitor treatment did not demonstrate an effect on Rck8 OPN-shRNA collagenase activity (Figure 6E). A similar pattern of collagenase activity was observed in OciLy3 nontarget and OPN-shRNA cells (Figure 6F).

## DISCUSSION

OPN is a multi-functional protein that plays an important role in various attributes of cancer including tumor growth, invasion, metastasis, and angiogenesis: and is frequently expressed in aggressive metastatic cancers, suggesting that it is a phenotypic marker of aggressiveness and high metastatic potential of cancer cells [12, 13, 30]. While we have discovered that OPN is the most upregulated gene in PCNSL [9], its role in this disease is not well characterized. In this study, we performed functional genomic experiments on OPN using B lymphoma cell models to determine its role in CNS lymphoma. We report for the first time that OPN plays an important role in CNS lymphoma. Our findings show that OPN promotes accelerated *in vitro* lymphoma cell proliferation and intracerebral tumor growth (Figures 1, 3). In addition, OPN enhances brain tissue invasiveness (Figures 2–4), and multidirectional intracerebral dissemination (Figure 4). In our preclinical models, increased OPN expression correlated with decreased survival, whereas OPN suppression significantly enhanced survival (Figure 3). Of note, manipulation of OPN expression in our study demonstrated changes in both secreted OPN (sOPN) and intracellular OPN (iOPN) levels (Figure 1). We have previously shown that intracellular OPN expression in PCNSL is predominantly nuclear [9], and validate this observation in the cell models used (Figures 1, 5). It has been previously shown in 293 cells that localization of iOPN in the nucleus is transient [31]. As such, it appears that the nuclear localization of iOPN is dependent on the cellular context.

Primary CNS lymphoma is an aggressive diffuse large B cell lymphoma which is described as “whole brain disease” [6] due to the invasion and widespread dissemination of lymphoma cells in the brain. This “whole brain” nature makes PCNSL difficult to cure and necessitates treatments that impact the entire central nervous system. The underlying molecular pathogenic mechanism of these biological traits must be better understood to develop more specific treatments for PCNSL. Our novel findings suggest that OPN plays a crucial multi-faceted role in the aggressive biological behavior and the “whole brain” nature of PCNSL and represents a novel therapeutic target.

The ability of OPN to activate a variety of downstream mediators dependent on NF-κB activation has been demonstrated previously [23, 32, 33], however the mechanism by which it does so has not been well defined, and additionally may vary according to cellular context. While OPN has been reported to affect NF-κB expression through a variety of indirect mechanisms such as through PI3K/AKT signaling [23], integrin signaling [25, 34], and CD44 activation of MAPK/P38 [35], the specific relationship whereby OPN facilitates this mediation has not been elucidated in PCNSL. Moreover, several reports suggest that expression of OPN may in turn be positively regulated by NF-κB, suggesting a positive feedback loop [36, 37].

**Figure 7:**
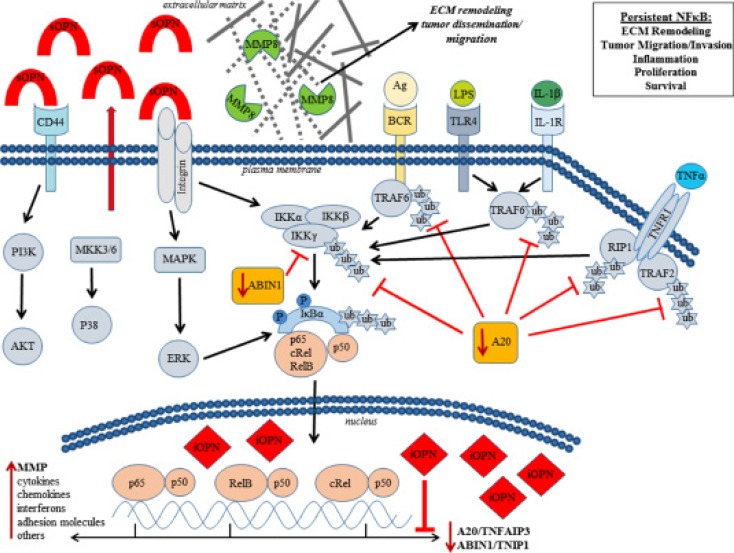
Osteopontin (OPN) activates NF-κB signaling via a novel dual mechanism involving secretory OPN (sOPN) and intracellular OPN (iOPN) sOPN induces NF-κB signaling by acting on cell surface receptors via an autocrine loop while iOPN activates NF-κB signaling by transcriptionally downregulating A20/TNFAIP3 and ABIN1/TNIP1. As A20 and ABIN1 are negative regulators responsible for terminating and limiting NF-κB signaling, their downregulation would have an amplifying and prolonging effect on NF-κB signaling by other inducers including sOPN. Our data show that OPN-induced activation of NF-κB results in increased tumor growth and activation of MMP8 leading to tumor invasion and dissemination.

Mechanistically, we show for the first time that OPN promotes NF-κB activation via a novel dual mechanism involving intracellular OPN (iOPN) and secretory OPN (sOPN). We find that iOPN activates NF-κB signaling by transcriptionally downregulating A20/TNFAIP3 and ABIN1/TNIP1 (Figure 5B–5E). As A20/TNFAP3 and ABIN1/TNIP1 are negative regulators responsible for terminating and limiting the NF-κB signaling [38, 39], their downregulation by iOPN would have an amplifying effect on activation of NF-κB by other inducers. Therefore, iOPN-expressing lymphoma cells will be susceptible to protracted and amplified NF-κB activity. On the other hand, sOPN induces NF-κB signaling by acting on cell surface receptors via an autocrine loop without significant change in A20/TNFAIP3 and ABIN1/TNIP1 in lymphoma cells (Figure 5F–5G). sOPN has previously been shown to activate NF-κB signaling via integrins [40]. As such, iOPN and sOPN, working together, can greatly enhance NF-κB activity. We have summarized our novel mechanistic findings in a diagram (Figure 7). Future experiments are needed to elucidate the specific mechanism by which iOPN influences the expression of A20/TNFAIP3 and ABIN1/TNIP1, with reference to PCNSL patient samples. As A20 has been shown to be regulated by microRNAs [41], the relationship between iOPN and microRNAs needs to be explored.

Our results show that OPN-mediated activation of NF-κB signaling is an important mechanism of the aggressive tumor growth, invasion, and widespread intracerebral dissemination of lymphoma cells in CNSL. OPN-mediated NF-κB activation is clearly linked to lymphoma cell proliferation (Figure 6A), thus establishing a signaling pathway for aggressive tumor growth in CNSL (OPN → NF-κB activation → tumor growth). Previous studies have found that OPN activates expression of MMP proteins [34, 42]. In this study, we found that MMP8 positively correlates with OPN expression (Figure 6B). Lymphoma cell invasion was attenuated with an MMP8 inhibitor, implicating OPN-induced expression of this particular collagenase for the first time in lymphoma cell invasion. Moreover, the NF-κB inhibitor (IKK2) similarly suppressed lymphoma cell invasiveness and collagenase activity (Figure 6E and 6F), supporting a direct role for OPN-mediated activation of NF-κB in MMP8 regulation, extracellular matrix (ECM) remodeling, and lymphoma cell invasion. As such, we have established a signaling pathway for the “whole brain” nature of CNSL (OPN → NF-κB activation → MMP8 → invasion and dissemination). MMP8 is reported to play multiple roles in the development and regulation of inflammatory disease [43]. Interestingly, MMP8 has been shown to facilitate blood brain barrier (BBB) disruption and increased permeability in bacterial meningitis by mediating proteolytic cleavage of the tight junction protein occludin, and detachment of human brain microvascular endothelial cells [44]. MMP8 expression also has been reported in other lymphomas such as mantle cell lymphoma and Waldenstrom macroglobulinemia and correlates with poor prognosis and lymphoma growth [45, 46]. Increased expression of OPN has also been reported in damaged tissue immediately surrounding blood vessels with BBB impairment [47]. Given this information, OPN directed MMP8 expression might be critical for highly selective CNS tropism and penetration of lymphoma cells. Our future plan is to correlate the expression of MMP8 with OPN expression in human PCNSL tissue and evaluate their role in selective CNS tropism and intracerebral dissemination of PCNSL using our models.

In conclusion, our results identify OPN as a multi-faceted protumorigenic driver, which facilitates tumor growth, invasion, and dissemination in CNS lymphoma. We propose OPN as a candidate for the development of targeted therapy for PCNSL. Additional validation of our findings in other CNS lymphoma models and human PCNSL tissue specimens is warranted.

## MATERIALS AND METHODS

### Reagents

RPMI-1640, FBS, and Pencillin/Streptomycin/Amphotercin B (PSA) were purchased from CellGro; IMDM medium was purchased from Thermo Scientific; OptiMEM was purchased from Invitrogen; Puromycin (Sigma-Aldrich, Inc.); Quantikine human osteopontin ELISA kit (R & D Systems, Inc.); Rabbit anti-osteopontin (Rockland Immunochemicals, Inc. Limerick, PA); molecular biology-grade reagents (Sigma); Trizol and PureLink RNA extraction kit (Life Technologies); Nano 6000 RNA analysis reagents (Agilent); PCR primers and TaqMan FAST Universal PCR Master Mix (Life Technologies); BD Biocoat Matrigel Invasion Chamber (BD Biosciences); Recombinant OPN (R & D Systems, Inc.).

### Tissues and cell lines

Raji, Rck8, and OCILY3 lymphoma cells were used in experiments. Raji cell line was purchased from American Tissue Culture. Rck8 cell are a generous gift by Dr. Izidore Lossos at University of Miami. OCILY3 cells are a generous gift by Dr. Arthur L. Shaffer at National Cancer Institute. OCILY3 cells were maintained in IMDM media with 20% (v/v) FBS and 1% (v/v) PSA. All other cell lines were maintained in RPMI-1640 media with L-glutamine and supplemented with 10% (v/v) FBS and 1% (v/v) PSA. Cells infected with lentivirus constructs were first grown in selection media containing 2.5 ug/ml of Puromycin for five days.

### Lentiviral plasmids and transfection of lymphoma cell lines

pcDNA-OPN-V5 was a gift from Steven Johnson (Addgene plasmid # 11617). pLenti plasmids containing OPN (SPP1) shRNA constructs were obtained from Sigma-Aldrich, Inc. The luciferase-expressing pSIN-luc vector, a generous gift of Dr. Y. Ikeda, Mayo Clinic Rochester, was used to infect Raji and Rck8 transfectants. Producer cells (293 FT) were grown overnight at 4 × 10^6^ cells per 10 cm plate in DMEM transfection media containing 2–4 mM L-glutamine, 1 mM sodium pyruvate, 0.1 mM non-essential amino acids, and 10% FBS. The next day cells were pelleted and re-suspended in 3 ml OptiMEM supplemented with 10% FBS. Three micrograms of pLenti plasmid was packaged with 9 μg ViraPower Packaging Mix in 1.5 ml OptiMEM, mixed with 36 ul Lipofectamine 2000, and transfected into cells overnight at 37°C in a humidified CO_2_ incubator. Supernatant from pelleted 293 FT cells was collected, filtered, aliquoted, and stored at −80°C. For infection of Raji and Rck8 cells with lentivirus containing OPN, shRNA-, or luciferase-expressing constructs, 1 ml virus supernatant was mixed with 4 ml host cell suspension and polybrene was added for a final concentration of 7.5 ug/ml. Cells were incubated overnight in a humidified CO_2_ incubator. The next day cells were pelleted and re-suspended in complete media to remove virus and polybrene particles. Luciferase expression was determined using Dual-Luciferase Reporter Assay System on a Veritas Microplate Luminometer (Promega). For overexpressing OPN in Raji cells, 2.5 ug of plasmid DNA was used per well of a 6-well plate and antibiotic (G418) at 1 mg/ml was used as selection agent.

### RNA isolation and qRT-PCR

Lymphoma cells were cultured at 1 × 10^6^ cells in 2 ml of media. Cells were harvested by gentle centrifugation, and total RNA was extracted by Trizol (Invitrogen) and purified by RNeasy Plus mini Kit (Qiagen). Residual genomic DNA contamination was removed by DNA-Free kit (Life Technologies). One microgram of total RNA was converted to cDNA using High Capacity Reverse Transcription kit (Life Technologies) and diluted with nuclease-free water. Real-time PCR was used to measure changes in SPP1 mRNA expression in all parent and transfected lymphoma cell lines. Five microliters of diluted RNA (cDNA) in 15 ul TaqMan FAST Universal PCR Master Mix containing PCR primers was assayed by real-time PCR on an Applied Biosystems 7900HT FAST Real-Time PCR system (Life Technologies). C_t_ values were determined by instrument software and relative SPP1 mRNA values were normalized to GAPDH. Significant difference of C_t_ values was determined using a two-tailed Student's *T*-test. The primers used for qPCR were GAPDH (Hs99999905_m1), A20 (Hs00234713_m1), ABIN1 (Hs00374581_m1), and OPN (Hs00959010_m1) (Life Technologies).

### Proliferation assays

Cells were seeded in triplicate at 10^4^ cells per ml per well of a 24-well tissue culture plate for six days. Each day three wells from each group were counted in 10 ml Isoton II on a Z1 Coulter Particle Counter (Beckman-Coulter Corp). Data were plotted over time and two-tailed student *T*-test was used to analyze statistical significance of the differences.

### Invasion assay

Invasiveness of lymphoma cells were evaluated in triplicate by transwell invasion assays using BD Matrigel Invasion Chamber 8-um pore-size (Corning). Five percent serum was used as a chemoattractant in the bottom chamber.

### ELISA for osteopontin

10^5^ Raji and Rck8 cells were seeded in 5 ml media in 6-well plates in triplicate and were grown for four days. Cells were harvested and pelleted. Duplicate four hundred microliters of media supernatant were assayed for Osteopontin by ELISA according to manufacturer's protocol (R & D Systems).

### Electrophoretic mobility shift assay (EMSA)

Nuclear fractionation was first performed on cells to be assayed. Cells were washed twice with ice-cold PBS and the lysed in 1 ml lysis buffer (10 mM HEPES pH 7.9, 10 mM KCl, 0.1 mM EDTA, 0.1 mM EGTA, 1 mM DTT, 1 mM PMSF). Lysates were incubated for 15 min on ice. 62.5 μl 10% NP-40 was added and samples were placed on a shaker (2 min at 4°C). Samples were centrifuged (1 min; room temperature; 13,000 rpm) and the nuclei pellet was re-suspended in 100 μl high salt buffer (20 mM HEPES pH 7.9, 4 M NaCl, 1 mM EDTA, 1 mM EGTA, 1 mM DTT, 1 mM PMSF) followed by rough shaking for 20 min at 4°C, and centrifugation (5 min; 4°C; 13,000 rpm). Supernatants were transferred to a new tube and protein concentration was determined. IRDye 700 NF-κB Consensus Oligonucleotide and Odyssey Infrared EMSA Kit were purchased from LI- COR Biosciences. For mobility shift assays, 5 μg of nuclear protein was incubated on ice in 20 μl of a buffer containing 10 mM HEPES (pH 7.5), 50 mM KCl, 0.1 mM EDTA, 1 mM dithiotreitol, 0.1% NP-40 and 0.05 mg/ml poly (dI-dC). Labeled probe was added 30 min before gel loading. Samples were resolved on a non-denaturing 5% polyacrylamide gel in 0.5x TBE. Imaging was performed on the Odyssey (LI-COR Biosciences) using the 700 nm channel.

### Western blotting

Cells were lysed with RIPA buffer (Thermo Scientific) containing 1× Halt protease inhibitor (Thermo Scientific). Proteins were extracted from whole cells extracts were resolved by sodium dodecyl sulfate-polyacrylamide gel electrophoresis (SDS-PAGE), and proteins were transferred onto PVDF membranes for western blot analysis. After transfer, the membrane was blocked in membrane blocking solution (Invitrogen) overnight at 4°C. Blot was incubated with primary antibody overnight at 4°C. The primary antibody was diluted in buffer according to manufacturer's specification. The primary antibody was detected by the appropriate horseradish peroxidase-conjugated secondary antibody and followed by incubation with Pierce ECL Western Blotting Substrate (Thermo Scientific). Osteopontin (ab8448), ABIN1/TNIP1 (ab130720), and A/20TNFAIP3 (ab92324) were purchased from Abcam. RelB (sc-226), p65 (sc-372), c-Rel (sc-71), and p50 (sc-7178) were purchased from Santa Cruz Biotechnology.

### Reverse transcription PCR (RT-PCR)

Cellular mRNA was isolated using RNeasy Plus Mini Kit (74134, Qiagen) according to the manufacturer's instructions, and was transcribed into cDNA using High Capacity cDNA Reverse Transcription Kit (4368814, Applied Biosystems). The resulting cDNA was used to perform PCR analysis using the primers described elsewhere [48].

### Cellular fractionation

A cell fractionation kit (Cell Signaling Technology) was used to collect whole cell lysate (WCL), cytoplasmic (cyto), and nuclear (nuc) fractions from specified cell populations. Briefly, OciLy3 cells were treated with 50 ng of recombinant TNFΑα (Fisher) or PBS control for 1.5 hours in regular growth media. Cells were counted using a Coulter Counter analyzer (Beckman). 5 × 10^6^ cells per group were collected, and cell fractionation was performed per manufacturer specifications.

### Collagenase assay

A matrigel/collagen mixture was prepared using growth factor reduced, phenol-free matrigel (Corning) and DQ^™^ collagen, type I, fluorescein conjugate (Life Technologies) at a 10:1 ratio, maintained on ice. 12-well glass bottom plates (Mat Tek) were coated with 80 μL of the basement membrane mixture, and were allowed to equilibrate at 37°C in humidified conditions with 5% CO_2_ for 1 hour to solidify. 1 × 10^3^ cells per group were suspended in 350μL of basement membrane solution and added per well. 3D cultures were allowed to equilibrate for 2 hours to solidify prior to adding media containing reduced serum (0.5%) and either DMSO control, MMP8 inhibitor I (10 μM, EMD Millipore), or IKK2 inhibitor IV (10 uM, EMD Millipore). After 48 hours, cells were carefully washed with PBS 3×, fixed in 4% paraformaldehyde for 30 minutes, washed 3× with PBS, and stained with Texas Red^®^-X Phalloidin and DAPI (Life Technologies) (fixation and staining performed at room temperature). Cells were imaged using a laser-scanning confocal microscope (LSM 510 META; Carl Zeiss). Dapi and Phalloidin staining were used to locate B cell lymphoma colonies. Enzyme-driven hydrolysis of collagen was characterized by positive fluorescent signal derived from fluorescein per individual cell/colony. Positive or negative fluorescein expression was manually quantitated.

### *Ex-vivo* brain slice assay

The invasive potential of the Raji and Rck8 lymphoma cell lines were assessed *ex vivo* using a modified mouse brain slice assay [22]. Transfected Raji lymphoma cells, Raji-OPN and empty vector control cells were first pre-transfected with luciferase (SP-DiI; Molecular Probes). Brain slices were made from two-month-old male C57BL/6J mice (sacrificed by CO2 inhalation) by coronally sectioning the brain into 1 mm slices at +2.00 to −1.00 bregma. Brain slices were placed aseptically onto transwell (8 μm pore size) membranes in six-well dishes, and culture media (DMEM, 10% FCS, 6.5 μg/ml glucose, 100 u/ml penicillin, 100 μg/ml streptomycin, and 2.5 μg/ml amphotericin B) were added into each well to a point just below the surface of a brain slice. Lymphoma cells were deposited onto caudate putamen of brain slice. Cells were allowed to attach to the brain surface for one hour before the media is adjusted to just cover the surface of the slice. After 5 days of incubation, the extent of lymphoma cell invasion into the brain slice was quantitated by monitoring bioluminescence activity in both brain slice and the media. 3D luminescent topography reconstruction of the brain slices was performed using DLIT 3D Reconstruction software (Perkin Elmer) to evaluate the depth of lymphoma cell invasion into the brain slice. CD20 IHC of formalin-fixed brain slices with quantitation of CD20+ cells was performed to verify the presence of lymphoma cells in the brain tissue.

### Intracerebral lymphoma cells implantation

The intracerebral injection of lymphoma cells and bioluminescence imaging have been previously described [49].

### Statistics and survival analysis

ANOVA was used to determine the statistical significance of the differences between experimental groups. Kaplan-Meier survival curves were generated using Prism4 software GraphPad Software) and the statistical difference between curves was derived with a log-rank test. *P* < 0.05 was considered significant.

## Abbreviations

OPNO: steopontin
CNSL: CNS lymphoma
DLBCL: diffuse large B cell lymphoma
